# Serological and molecular investigation of hepatitis E virus in pigs reservoirs from Cameroon reveals elevated seroprevalence and presence of genotype 3

**DOI:** 10.1371/journal.pone.0229073

**Published:** 2020-02-10

**Authors:** Abdou Fatawou Modiyinji, Georges Marc Arthur Mveng Sanding, Marie Amougou Atsama, Chavely Gwladys Monamele, Moise Nola, Richard Njouom

**Affiliations:** 1 Department of Virology, Centre Pasteur of Cameroon, Yaoundé, Cameroon; 2 Department of Animals Biology and Physiology, Faculty of Sciences, University of Yaoundé I, Yaoundé, Cameroon; 3 Ministry of Livestock, Fisheries and Animal Industries, Yaoundé, Cameroon; CEA, FRANCE

## Abstract

**Background:**

Hepatitis E virus (HEV) is a zoonotic pathogen of which pigs have been established as reservoirs. In the present study, we investigated the presence of HEV among pigs in the Center and Littoral regions of Cameroon and performed the molecular characterization of positive strains.

**Methodology:**

A total of 453 serum and stool samples were randomly collected from pigs in slaughterhouses in Obala, Douala and Yaounde. All samples were examined for the presence of anti-HEV IgG and IgM antibodies using ELISA assays. IgM positive stool samples were tested for HEV RNA using an RT-PCR assay, followed by a nested PCR assay for sequencing and phylogenetic analysis.

**Results:**

Overall, 216 samples (47.7%, 95% CI: 43.1%-52.3%) were positive for at least one of the serological markers of HEV infection. Amongst these, 21.0% were positives for anti-HEV IgM, 17.7% for anti-HEV IgG, and 9.1% for both. A total of eight stool samples (5.9%) were positive for HEV RNA by nested RT-PCR. Phylogenetic analysis showed that the retrieved sequences clustered within HEV genotype 3.

**Conclusion:**

This study shows a high prevalence of anti-HEV antibodies and the circulation of genotype 3 in the swine population in Cameroon. Subsequent studies will be needed to elucidate the zoonotic transmission of HEV from pigs to humans in Cameroon.

## Introduction

Hepatitis E virus (HEV) is an emerging and zoonotic pathogen of humans and animals, which is responsible for significant morbidity and mortality especially in developing countries [1]. HEV affects approximately 20 million persons annually worldwide, causing over70.000 deaths [2].

HEV is a spherical, non-enveloped, single-stranded ribonucleic acid (RNA) virus belonging to the Hepeviridae family that contains several viral species divided into two genera: *Orthohepevirus* with four species (Orthohepevirus A–D) and *Piscihepevirus* with one species (Piscihepevirus A) [3]. Eight genotypes exist within *Orthohepevirus A* and these HEV strains infect humans and multiple mammals’ species. Genotype 1 and 2 are restricted to humans; genotype 3 is found among humans, swine, rabbits, deer and mongooses; genotype 4circulates between humans and swine; genotype 5 and 6 are found in wild boars; and genotype 7 and 8were recently identified in dromedary and Bactrian camels, respectively [4].

Several transmission routes of hepatitis E have been identified, and include: contamination by drinking infected water [5]; food-borne transmission by ingestion of meat from infected animals [6], transmission through milk consumption [7] and transfusion of infected blood products [8]. In developed countries, HEV is predominantly transmitted by the ingestion of pork and wild boar meat. Evidence for transmission of HEV-3 and HEV-4 by direct contact of humans with animals has been repeatedly described. Several studies have shown that persons with occupational contact to domestic pigs such as slaughterers, pig farmers or veterinarians exhibit significant higher anti-HEV antibody prevalence than the general population [9,10].

In Africa, contaminated water causes serious epidemic outbreaks. Other sources of infection such as animal transmission cannot be excluded since genotype 3 responsible for the zoonotic transmission of HEV has already been reported in some African countries [9,10]. The information on HEV infection in animals in this continent remains underreported [10]. In Cameroon, minimal attention has been paid to HEV epidemiology in human and animal populations. The aim of this study is to determine the seroprevalence of HEV infection in pigs in Center and Littoral regions of Cameroon and to determine the molecular characterization of identified viruses.

## Materials and methods

### Specimen collection

Fecal and serum samples were collected from 453 animals from domestic pigs in slaughterhouse in Obala, Douala and Yaounde, between February 2017 to September 2018. Cameroon has ten administrative regions including the littoral region where Douala is located and the central region where Yaoundé and Obala are located. Blood samples were collected at the bleeding post and the fecal samples were obtained and kept in sterile containers. All biological samples were transported in a cool box at 4°C, then frozen and stored at -80°C. Data such as age, sex, city and year of collection were recorded in a sheet dedicated to each pig. This study was approved by the Cameroonian Ministry of Livestock, Fisheries and Animal Industries (N˚000050/L/MINEPIA/SG/DREPIA/CE).

### ELISA for anti-HEV in swine

All sera were tested for the presence of anti-HEV immunoglobulins (Ig) with enzyme-linked immunosorbent assays: HEV IgG ELISA and HEV IgM ELISA 3.0 kits (MP Biomedicals Asia Pacific Pte Ltd, Singapore, formerly Genelabs Diagnostics Pte). The test was carried out according to the manufacturer's instructions by changing the human conjugates by porcine conjugates. Briefly, the sera diluted in diluent buffer were placed in coated wells. After incubation of 30 min at 37°C, followed by washing, the horseradish peroxidase (HRP)-conjugated goat anti-porcine IgM for IgM detection and goat anti-porcine IgG for IgG detection (Bethyl Laboratories Inc., USA) were added and incubated for 30 min at 37°C. Plates were washed, and 100 μl of substrate solution (tetramethylbenzidine) were added. The reaction was stopped after 15 min with 50 μl of stop solution (hydrochloric acid) and absorbance was measured at 450 nm using a spectrophotometer. Swine sera positive and negative for HEV were identified using positives and negatives controls provided with the kits. The blank and the positives controls have been assayed in duplicate, whereas the negatives controls in triplicate on each plate with each run of specimens. The manipulations were validated when the blank wells have an absorbance ≤ 0.100. Negatives controls have an absorbance ≤ 0.100 and the positives controls have absorbance ≥ 0.500 (For IgG detection) and ≥ 0.400 (For IgM detection) after substracting the blank.

### RNA extraction

Fecal samples were diluted at 10% (w/v) in phosphate-buffered saline, pH 7.2, clarified by centrifugation at 12,000g for 10 min and 140 ml aliquots of the clarified material was used for viral RNA extraction. RNA was extracted from 140 μl of suspension using a QIAamp Viral RNA Mini Kit (Qiagen, Courtaboeuf, France) according to the manufacturer’s instructions, eluted in 60 μl of RNase-free water, and stored at -80 C or used immediately.

### Amplification and sequencing of HEV

Fecal samples from pigs positive for anti-HEV antibodies were selected for amplification of a portion of ORF 2 targeting 731 bp by RT-PCR followed by nested PCR using a pair of primers as previously reported by Huang et al. [11]. The RT-PCR was performed using SuperScript^™^ III One-Step RT-PCR System with Platinum Taq (Life Technologies Corporation, USA) in a final volume of 50 μL containing 0.2 μM of each primer (3156N and 3157N), 2.3 mM MgSO4, 200 μM dNTPs and 10 μL of RNA extract. A Perkin Elmer Gene Amp PCR System 9700 was used with the following cycling conditions: 50°C for 30 min, 94°C for 2 min, 40 cycles of 94°C for 15 s, 60°C for 30 s, and 72°C for 1 min, and a final elongation step at 72°C for 5 min. In the nested PCR, 5 μL of RT-PCR products were used in a final volume of 50 μL containing 1.25 U of Taq polymerase (Life Technologies Corporation, USA), 0.2 μM of each primer (3158N and 3159N), and 1.5 mM MgCl2. The cycling conditions consisted of 94°C for 5 min, 40 cycles at 94°C for 30 s, 55°C for 30 s, and 72°C for 1 min 30 s, and a final elongation step at 72°C for 10 min. The 348 nucleotides (nt) second round PCR products were visualized after electrophoresis on a 1.5% agarose gel. The nested PCR products were further sequenced on the 3730XL DNA Analyzer (Applied Biosystems), by Sanger method using GenomeLab DTCS-Quick Start Kit (Beckman Coulter, Paris, France) according to the manufacturer’s instructions. Phylogenetic analysis was performed by comparing Cameroonian strains with the selected strains of various geographical origins obtained from GenBank. Phylogenetic trees were constructed using the Kimura two‐parameter model and neighbour‐joining method component of the Molecular Evolutionary Genetics Analysis (MEGA) 6 program on the basis of the nucleotide sequences of the amplified gene. Robustness of the tree topologies was estimated by bootstrap analysis with 1000 pseudo-replicate data sets, and only bootstrap values > 70 were considered significant.

### Statistical analysis

Prevalence is given as percentages and categorical variables were compared applying Fisher's exact or chi‐square tests. The level of significance was *P* < 0.05. All the analyses were performed using SPSS 16.0 statistical software.

## Results

### Prevalence of anti-HEV antibodies among pigs

A total of 453 plasmas were collected and tested for the presence of anti-HEV antibodies. Overall, 216 samples (34.9%, 95% CI: 31.8%-38.1%) were positive for at least one of the serological markers of HEV infection. Amongst these, 21.0% (95/453) were positives for anti-HEV IgM, 17.7% (80/453) for anti-HEV IgG, and 9.1% (41/453) for both (Table 1).

**Table 1:** Results of detection of specific anti-hepatitis E virus antibodies.

	Yaounde n (%)	Douala n (%)	Obala n (%)	Total N (%)
No. participants	303	102	48	453
**Anti-IgM and/or IgG positive**	176 (58.1)	26 (25.5)	14 (29.2)	216 (47.7)
**Anti-IgM positive and IgG negative**	76 (25.1)	5 (4.9)	14 (29.2)	95 (21.0)
**Anti-IgG positive and IgM negative**	62 (20.5)	18 (17.6)	0	80 (17.7)
**Anti-IgM and IgG positives**	38 (12.5)	3 (2.9)	0	41 (9.1)
**Anti-IgM and IgG negatives**	127 (41.9)	76 (74.5)	34 (70.8)	237 (52.3)

IgG: Immunoglobulin G, IgM: Immunoglobulin M, No.: number

Within the pigs collected in Yaounde, 58.1% (176/303) were positive for at least one serological marker. Of these, the percentage of IgM positivity was 25.1% (76/303); meanwhile 20.5% (62/303) were positives to anti-HEV IgG and 12.5% (38/303) had both markers (Table 2). In Douala, 25.5% (26/102) of samples were positive for at least one serological marker. Of these, the percentage of IgM positivity was 4.9% (5/102); meanwhile 17.6% (18/102) were seropositive to anti-HEV IgG and 2.9% (3/102) had both markers. In Obala, 14 out of 48 samples (29.2%) were IgM positive and no samples were positives for IgG. Higher anti-HEV positivity was noted in 2018 (25.1% for IgM, 35.2% for IgG and 12.5% for both antibodies) than in 2017 (12.7% for IgM, 12% for IgG and 2% for both markers), with a statistically significant difference (P<0.05). The prevalence of IgG in pigs in Douala (17.6%) and Yaounde (20.5%) is statistically higher than that found in pigs taken from Obala (0%) (p = 0.003). However, the prevalence of IgM in pigs collected in Obala (29.2%) is statistically higher than that found in Douala (4.9%) and Yaounde (25.1%) (P<0.001). No difference was found between HEV seropositivity, sex and age of pigs collected (P> 0.05) (Table 2).

**Table 2:** Seroprevalence of HEV infection by different factors.

	Tested pigs N	IgG positives, n (%)	P-value	IgM positives, n (%)	P-value	IgG and IgM positives, n (%)	P-value
**Age**							
<6 months	79	17 (21.5)	0.3	22 (27.8)	0.1	9 (11.4)	0.4
>6 months	374	63 (16.8)		73 (19.5)		32 (8.6)	
**Year**							
2017	150	18 (12.0)	0.03	19 (12.7)	0.002	3 (2.0)	<0.001
2018	303	62 (35.2)		76 (25.1)		38 (12.5)	
**Sex**							
Male	183	33 (18.0)	0.9	42 (23.0)	0.4	13 (7.1)	0.2
Female	270	47 (17.4)		53 (19.6)		28 (10.4)	
**City**							
Douala	102	18 (17.6)	0.003	5 (4.9)	<0.001	3 (2.9)	0.001
Yaounde	303	62 (20.5)		76 (25.1)		38 (12.5)	
Obala	48	0		14 (29.2)		0	

IgG: Immunoglobulin G, IgM: Immunoglobulin M, n: Number positive, N: Number tested

### Prevalence of HEV RNA

A total of 136 stool samples collected from pigs which were serologically tested positive in anti-HEV IgM were selected and processed in nested RT-PCR. Of these, 8 (5.9%) samples were found positive for HEV viral RNA. All the positive samples came from stool samples taken from pigs in Yaounde.

### Genotyping and phylogenetic analysis of HEV

**Fig 1:**
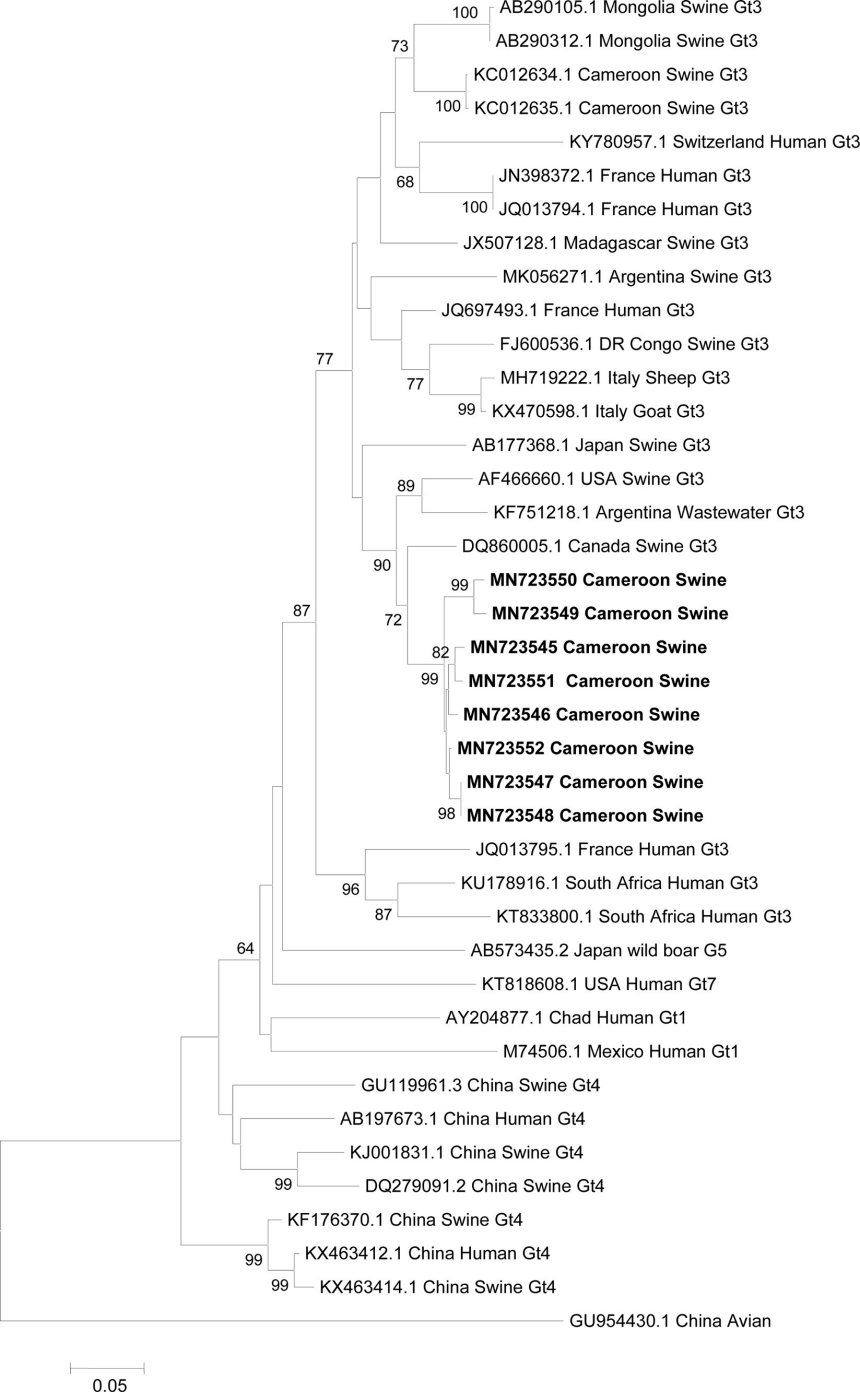
Phylogenetic tree based on a 348-bp fragment of the ORF2 region of 32 reference sequences retrieved from Genbank. The neighbor-joining tree was drawn using MEGA 6 software, using the Kimura two‐parameter model as a correction factor and 1000 replicates. Bootstrap values >70 are shown. Strains identified in this study are shown in bold. Reference strains from GenBank are included, and for all entries, the accession number, country of origin, host and genotype are reported.

The nucleotide sequences of HEV strains isolated in this study have been deposited in GenBank with the accession numbers (MN723545, MN723546, MN723547, MN723548, MN723549, MN723550, MN723551 and MN723552). Phylogenetic analysis of the eight isolates of swine HEV was carried out by comparing the highly preserved 348 bp of OFR 2 to HEV references strains from different geographical regions contained in GenBank. Phylogenetic analysis showed that all eight Cameroon strains belonged to genotype 3 and clustered with human, goat, sheep and swine HEV strains from different geographical regions of the world especially with the swine strains from Canada and USA and Argentina Wastewater HEV strains (Fig 1).

## Discussion

There is accumulated evidence that HEV is enzootic in a broad range of animals in Cameroon. Serological analyses have revealed the presence of anti-HEV antibodies among pigs and non-human primates in previous studies in Cameroon [12,13]. We noted high anti-HEV seropositivity in this study of 47.7% similarly to the 43.2% that was previously reported in the North and West regions of Cameroon [12], confirming that pigs are indeed a main reservoir for HEV. We noted anti-HEV IgM and IgG seropositivity of 21.0% and 17.7% respectively which is higher than reports from Nigeria and Mexico and lower than reports from Madagascar and Scotland [2,10,14,15]. These differences could be explained, at least partly, by the different pig husbandry systems, the routine management and hygiene practices applied on these pig farms and environmental conditions that characterize various areas [16]. This difference may also be associated with the different HEV diagnostic methods used in these different studies. Our study shows high seroprevalence of IgM (21.0%) compared to IgG (17.7%) similarly to the previous study by Modiyinji et al. [12]. This could be explained by the fact that majority of the pigs in this study were older than 6 months, since, Seminati *et al*. in 2008 shows that pigs older than 12 weeks of age have a higher IgM seroprevalence, compared to IgG seroprevalence [17].This high prevalence of IgM compared to IgG could be explained by the fact that Obala pigs were positive only for IgM antibodies while in Yaounde and Douala, both types of antibodies were detected. The presence of IgM antibodies alone in pigs sampled at Obala suggests an epidemic of HEV in this pig population. Poor conditioning of the samples would have prevented the detection of RNA in the samples of these pigs. Published studies have shown that humans and other animals including pigs transmit maternal IgG across the placenta and this may provide protection for some time, and may contribute to antibody class combinations being different depending on age [18]. There was no significant difference between male and female (P> 0.05), similarly to reports from Madagascar in 2013 [10]. Our study shows a significant difference between the spatial distributions of swine HEV seroprevalences (P value < 0.05).

HEV RNA was detected in a small proportion (5.9%) of stool samples from pigs that tested seropositive for anti-HEV IgM, all of which were from pigs in Yaounde. In agreement with distance analysis, phylogenetic reconstruction using partial nucleotide sequences of ORF 2 (348 nt) showed a close relationship of our strains with HEV genotype 3 strains. A previous study conducted in Cameroon in 2013 had already shown the presence of genotype 3 in the liver samples of pigs in Yaounde [19]. Although the absence of animal traceability in Cameroon excludes the possibility of the identification of an individual pig’s farm at source, we can say without reserve that HEV circulates in the pig’s population in Yaounde. Phylogenetic analysis showed that swine strains from Cameroon are very close to swine strains from Canada and USA. However, more swine and human HEV sequences are required to shed light the origin of HEV strains in the swine population and the possibility of a swine-to-human transmission of HEV. Previous studies have identified workers in slaughterhouses and pig handlers as populations at high risk of infection, because of their frequent contacts with organs, manure, and blood from animals [20,21]. Further studies in these high risk populations will be needed to fully elucidate the zoonotic route of HEV in Cameroon.

## Conclusion

This study showed the presence of anti-HEV antibodies in pigs in the littoral and Centre regions of Cameroon and the circulation of genotype 3 in a swine population of Yaounde. Subsequent studies will be needed to elucidate the possibility of a zoonotic transmission of HEV from pigs to humans in Cameroon.

## Supporting information

S1 Database (PDF)
