# Supplementary material for: Serological and molecular investigation of hepatitis E virus in pigs reservoirs from Cameroon reveals elevated seroprevalence and presence of genotype 3
Source: PLoS One. 2020 Feb 10;15(2):e0229073. doi: 10.1371/journal.pone.0229073 (PMC7010275; doi:10.1371/journal.pone.0229073)
Supplement: S1 Database — (PDF) [file pone.0229073.s001.pdf]

Année	Sexe	Age	région	Ville	IgG HEV	IgM HEV	IgG et/ou IgM	IgG et IgM	RT-PCR nichée
2017	M	plus de 6 mois	Centre	Obala	Négatif	Négatif	Négatif	Négatif	Négatif
2017	M	plus de 6 mois	Centre	Obala	Négatif	Positif	Positif	Négatif	Négatif
2017	M	plus de 6 mois	Centre	Obala	Négatif	Négatif	Négatif	Négatif	Négatif
2017	M	plus de 6 mois	Centre	Obala	Négatif	Négatif	Négatif	Négatif	Négatif
2017	M	plus de 6 mois	Centre	Obala	Négatif	Négatif	Négatif	Négatif	Négatif
2017	M	plus de 6 mois	Centre	Obala	Négatif	Négatif	Négatif	Négatif	Négatif
2017	M	plus de 6 mois	Centre	Obala	Négatif	Négatif	Négatif	Négatif	Négatif
2017	M	plus de 6 mois	Centre	Obala	Négatif	Négatif	Négatif	Négatif	Négatif
2017	M	plus de 6 mois	Centre	Obala	Négatif	Positif	Positif	Négatif	Négatif
2017	M	plus de 6 mois	Centre	Obala	Négatif	Positif	Positif	Négatif	Négatif
2017	F	plus de 6 mois	Centre	Obala	Négatif	Positif	Positif	Négatif	Négatif
2017	F	plus de 6 mois	Centre	Obala	Négatif	Négatif	Négatif	Négatif	Négatif
2017	F	plus de 6 mois	Centre	Obala	Négatif	Négatif	Négatif	Négatif	Négatif
2017	M	plus de 6 mois	Centre	Obala	Négatif	Négatif	Négatif	Négatif	Négatif
2017	M	plus de 6 mois	Centre	Obala	Négatif	Positif	Positif	Négatif	Négatif
2017	M	plus de 6 mois	Centre	Obala	Négatif	Négatif	Négatif	Négatif	Négatif
2017	F	plus de 6 mois	Centre	Obala	Négatif	Positif	Positif	Négatif	Négatif
2017	F	plus de 6 mois	Centre	Obala	Négatif	Positif	Positif	Négatif	Négatif
2017	F	plus de 6 mois	Centre	Obala	Négatif	Négatif	Négatif	Négatif	Négatif
2017	F	plus de 6 mois	Centre	Obala	Négatif	Négatif	Négatif	Négatif	Négatif
2017	F	plus de 6 mois	Centre	Obala	Négatif	Négatif	Négatif	Négatif	Négatif
2017	M	plus de 6 mois	Centre	Obala	Négatif	Positif	Positif	Négatif	Négatif
2017	F	plus de 6 mois	Centre	Obala	Négatif	Positif	Positif	Négatif	Négatif
2017	F	plus de 6 mois	Centre	Obala	Négatif	Négatif	Négatif	Négatif	Négatif
2017	F	plus de 6 mois	Centre	Obala	Négatif	Négatif	Négatif	Négatif	Négatif
2017	M	plus de 6 mois	Centre	Obala	Négatif	Négatif	Négatif	Négatif	Négatif
2017	F	plus de 6 mois	Centre	Obala	Négatif	Négatif	Négatif	Négatif	Négatif
2017	M	plus de 6 mois	Centre	Obala	Négatif	Négatif	Négatif	Négatif	Négatif
2017	M	moins de 6 mois	Centre	Obala	Négatif	Positif	Positif	Négatif	Négatif
2017	M	moins de 6 mois	Centre	Obala	Négatif	Négatif	Négatif	Négatif	Négatif
2017	M	moins de 6 mois	Centre	Obala	Négatif	Positif	Positif	Négatif	Négatif
2017	F	plus de 6 mois	Centre	Obala	Négatif	Négatif	Négatif	Négatif	Négatif
2017	M	plus de 6 mois	Centre	Obala	Négatif	Négatif	Négatif	Négatif	Négatif
2017	F	moins de 6 mois	Centre	Obala	Négatif	Négatif	Négatif	Négatif	Négatif
2017	F	moins de 6 mois	Centre	Obala	Négatif	Négatif	Négatif	Négatif	Négatif
2017	M	plus de 6 mois	Centre	Obala	Négatif	Négatif	Négatif	Négatif	Négatif
2017	M	plus de 6 mois	Centre	Obala	Négatif	Négatif	Négatif	Négatif	Négatif
2017	M	plus de 6 mois	Centre	Obala	Négatif	Positif	Positif	Négatif	Négatif
2017	M	plus de 6 mois	Centre	Obala	Négatif	Négatif	Négatif	Négatif	Négatif
2017	M	plus de 6 mois	Centre	Obala	Négatif	Négatif	Négatif	Négatif	Négatif
2017	M	plus de 6 mois	Centre	Obala	Négatif	Négatif	Négatif	Négatif	Négatif
2017	M	plus de 6 mois	Centre	Obala	Négatif	Positif	Positif	Négatif	Négatif
2017	M	plus de 6 mois	Centre	Obala	Négatif	Positif	Positif	Négatif	Négatif

2017	F	plus de 6 mois	Centre	Obala	Négatif	Négatif	Négatif	Négatif	Négatif
2017	M	plus de 6 mois	Centre	Obala	Négatif	Négatif	Négatif	Négatif	Négatif
2017	F	plus de 6 mois	Centre	Obala	Négatif	Négatif	Négatif	Négatif	Négatif
2017	M	plus de 6 mois	Centre	Obala	Négatif	Négatif	Négatif	Négatif	Négatif
2017	F	plus de 6 mois	Centre	Obala	Négatif	Négatif	Négatif	Négatif	Négatif
2017	F	moins de 6 mois	Littoral	Douala	Négatif	Négatif	Négatif	Négatif	Négatif
2017	F	plus de 6 mois	Littoral	Douala	Négatif	Négatif	Négatif	Négatif	Négatif
2017	F	plus de 6 mois	Littoral	Douala	Positif	Négatif	Positif	Négatif	Négatif
2017	M	plus de 6 mois	Littoral	Douala	Positif	Négatif	Positif	Négatif	Négatif
2017	M	moins de 6 mois	Littoral	Douala	Négatif	Négatif	Négatif	Négatif	Négatif
2017	F	plus de 6 mois	Littoral	Douala	Négatif	Négatif	Négatif	Négatif	Négatif
2017	F	plus de 6 mois	Littoral	Douala	Négatif	Positif	Positif	Négatif	Négatif
2017	M	plus de 6 mois	Littoral	Douala	Positif	Positif	Positif	Positif	Négatif
2017	M	plus de 6 mois	Littoral	Douala	Négatif	Négatif	Négatif	Négatif	Négatif
2017	F	plus de 6 mois	Littoral	Douala	Négatif	Négatif	Négatif	Négatif	Négatif
2017	F	moins de 6 mois	Littoral	Douala	Négatif	Négatif	Négatif	Négatif	Négatif
2017	F	plus de 6 mois	Littoral	Douala	Négatif	Négatif	Négatif	Négatif	Négatif
2017	M	moins de 6 mois	Littoral	Douala	Négatif	Négatif	Négatif	Négatif	Négatif
2017	F	plus de 6 mois	Littoral	Douala	Négatif	Négatif	Négatif	Négatif	Négatif
2017	F	plus de 6 mois	Littoral	Douala	Négatif	Négatif	Négatif	Négatif	Négatif
2017	F	plus de 6 mois	Littoral	Douala	Négatif	Négatif	Négatif	Négatif	Négatif
2017	F	moins de 6 mois	Littoral	Douala	Négatif	Positif	Positif	Négatif	Négatif
2017	F	plus de 6 mois	Littoral	Douala	Positif	Négatif	Positif	Négatif	Négatif
2017	F	plus de 6 mois	Littoral	Douala	Positif	Négatif	Positif	Négatif	Négatif
2017	M	moins de 6 mois	Littoral	Douala	Positif	Négatif	Positif	Négatif	Négatif
2017	M	plus de 6 mois	Littoral	Douala	Négatif	Négatif	Négatif	Négatif	Négatif
2017	M	plus de 6 mois	Littoral	Douala	Négatif	Négatif	Négatif	Négatif	Négatif
2017	F	plus de 6 mois	Littoral	Douala	Négatif	Négatif	Négatif	Négatif	Négatif
2017	F	plus de 6 mois	Littoral	Douala	Négatif	Négatif	Négatif	Négatif	Négatif
2017	F	plus de 6 mois	Littoral	Douala	Négatif	Négatif	Négatif	Négatif	Négatif
2017	F	plus de 6 mois	Littoral	Douala	Négatif	Négatif	Négatif	Négatif	Négatif
2017	F	plus de 6 mois	Littoral	Douala	Positif	Négatif	Positif	Négatif	Négatif
2017	M	plus de 6 mois	Littoral	Douala	Négatif	Négatif	Négatif	Négatif	Négatif
2017	M	plus de 6 mois	Littoral	Douala	Négatif	Négatif	Négatif	Négatif	Négatif
2017	F	plus de 6 mois	Littoral	Douala	Négatif	Négatif	Négatif	Négatif	Négatif
2017	M	plus de 6 mois	Littoral	Douala	Négatif	Négatif	Négatif	Négatif	Négatif
2017	M	plus de 6 mois	Littoral	Douala	Négatif	Négatif	Négatif	Négatif	Négatif
2017	M	plus de 6 mois	Littoral	Douala	Négatif	Négatif	Négatif	Négatif	Négatif
2017	F	plus de 6 mois	Littoral	Douala	Négatif	Négatif	Négatif	Négatif	Négatif
2017	F	plus de 6 mois	Littoral	Douala	Négatif	Négatif	Négatif	Négatif	Négatif
2017	M	plus de 6 mois	Littoral	Douala	Négatif	Négatif	Négatif	Négatif	Négatif
2017	M	moins de 6 mois	Littoral	Douala	Négatif	Négatif	Négatif	Négatif	Négatif
2017	M	plus de 6 mois	Littoral	Douala	Négatif	Négatif	Négatif	Négatif	Négatif
2017	M	plus de 6 mois	Littoral	Douala	Négatif	Négatif	Négatif	Négatif	Négatif

2017	F	plus de 6 mois	Littoral	Douala	Négatif	Négatif	Négatif	Négatif	Négatif
2017	F	plus de 6 mois	Littoral	Douala	Négatif	Négatif	Négatif	Négatif	Négatif
2017	M	plus de 6 mois	Littoral	Douala	Négatif	Négatif	Négatif	Négatif	Négatif
2017	F	plus de 6 mois	Littoral	Douala	Négatif	Négatif	Négatif	Négatif	Négatif
2017	M	plus de 6 mois	Littoral	Douala	Positif	Négatif	Positif	Négatif	Négatif
2017	M	plus de 6 mois	Littoral	Douala	Positif	Négatif	Positif	Négatif	Négatif
2017	M	plus de 6 mois	Littoral	Douala	Négatif	Négatif	Négatif	Négatif	Négatif
2017	F	plus de 6 mois	Littoral	Douala	Négatif	Négatif	Négatif	Négatif	Négatif
2017	M	plus de 6 mois	Littoral	Douala	Négatif	Négatif	Négatif	Négatif	Négatif
2017	M	plus de 6 mois	Littoral	Douala	Négatif	Positif	Positif	Négatif	Négatif
2017	F	plus de 6 mois	Littoral	Douala	Négatif	Négatif	Négatif	Négatif	Négatif
2017	M	plus de 6 mois	Littoral	Douala	Positif	Négatif	Positif	Négatif	Négatif
2017	M	plus de 6 mois	Littoral	Douala	Négatif	Négatif	Négatif	Négatif	Négatif
2017	F	plus de 6 mois	Littoral	Douala	Négatif	Négatif	Négatif	Négatif	Négatif
2017	F	plus de 6 mois	Littoral	Douala	Négatif	Négatif	Négatif	Négatif	Négatif
2017	F	plus de 6 mois	Littoral	Douala	Positif	Négatif	Positif	Négatif	Négatif
2017	F	plus de 6 mois	Littoral	Douala	Négatif	Négatif	Négatif	Négatif	Négatif
2017	M	plus de 6 mois	Littoral	Douala	Positif	Négatif	Positif	Négatif	Négatif
2017	F	plus de 6 mois	Littoral	Douala	Négatif	Négatif	Négatif	Négatif	Négatif
2017	F	plus de 6 mois	Littoral	Douala	Négatif	Négatif	Négatif	Négatif	Négatif
2017	F	plus de 6 mois	Littoral	Douala	Négatif	Négatif	Négatif	Négatif	Négatif
2017	M	plus de 6 mois	Littoral	Douala	Négatif	Négatif	Négatif	Négatif	Négatif
2017	F	plus de 6 mois	Littoral	Douala	Négatif	Négatif	Négatif	Négatif	Négatif
2017	F	plus de 6 mois	Littoral	Douala	Négatif	Négatif	Négatif	Négatif	Négatif
2017	M	plus de 6 mois	Littoral	Douala	Négatif	Négatif	Négatif	Négatif	Négatif
2017	M	moins de 6 mois	Littoral	Douala	Négatif	Négatif	Négatif	Négatif	Négatif
2017	F	plus de 6 mois	Littoral	Douala	Négatif	Négatif	Négatif	Négatif	Négatif
2017	F	plus de 6 mois	Littoral	Douala	Négatif	Négatif	Négatif	Négatif	Négatif
2017	F	plus de 6 mois	Littoral	Douala	Négatif	Négatif	Négatif	Négatif	Négatif
2017	F	moins de 6 mois	Littoral	Douala	Négatif	Négatif	Négatif	Négatif	Négatif
2017	F	plus de 6 mois	Littoral	Douala	Négatif	Négatif	Négatif	Négatif	Négatif
2017	M	moins de 6 mois	Littoral	Douala	Positif	Négatif	Positif	Négatif	Négatif
2017	F	plus de 6 mois	Littoral	Douala	Négatif	Négatif	Négatif	Négatif	Négatif
2017	F	plus de 6 mois	Littoral	Douala	Négatif	Négatif	Négatif	Négatif	Négatif
2017	F	plus de 6 mois	Littoral	Douala	Négatif	Négatif	Négatif	Négatif	Négatif
2017	F	plus de 6 mois	Littoral	Douala	Positif	Négatif	Positif	Négatif	Négatif
2017	M	plus de 6 mois	Littoral	Douala	Positif	Positif	Positif	Positif	Négatif
2017	F	plus de 6 mois	Littoral	Douala	Négatif	Négatif	Négatif	Négatif	Négatif
2017	F	plus de 6 mois	Littoral	Douala	Négatif	Positif	Positif	Négatif	Négatif
2017	F	plus de 6 mois	Littoral	Douala	Négatif	Négatif	Négatif	Négatif	Négatif
2017	F	plus de 6 mois	Littoral	Douala	Négatif	Négatif	Négatif	Négatif	Négatif
2017	F	moins de 6 mois	Littoral	Douala	Négatif	Positif	Positif	Négatif	Négatif
2017	M	plus de 6 mois	Littoral	Douala	Négatif	Négatif	Négatif	Négatif	Négatif
2017	F	plus de 6 mois	Littoral	Douala	Positif	Négatif	Positif	Négatif	Négatif

2017	M	moins de 6 mois	Littoral	Douala	Positif	Positif	Positif	Positif	Négatif
2017	F	plus de 6 mois	Littoral	Douala	Négatif	Négatif	Négatif	Négatif	Négatif
2017	M	plus de 6 mois	Littoral	Douala	Négatif	Négatif	Négatif	Négatif	Négatif
2017	F	plus de 6 mois	Littoral	Douala	Négatif	Négatif	Négatif	Négatif	Négatif
2017	F	plus de 6 mois	Littoral	Douala	Négatif	Négatif	Négatif	Négatif	Négatif
2017	M	plus de 6 mois	Littoral	Douala	Négatif	Négatif	Négatif	Négatif	Négatif
2017	F	plus de 6 mois	Littoral	Douala	Négatif	Négatif	Négatif	Négatif	Négatif
2017	F	plus de 6 mois	Littoral	Douala	Négatif	Négatif	Négatif	Négatif	Négatif
2017	M	plus de 6 mois	Littoral	Douala	Négatif	Négatif	Négatif	Négatif	Négatif
2017	M	moins de 6 mois	Littoral	Douala	Positif	Négatif	Positif	Négatif	Négatif
2017	F	plus de 6 mois	Littoral	Douala	Négatif	Négatif	Négatif	Négatif	Négatif
2017	F	plus de 6 mois	Littoral	Douala	Positif	Négatif	Positif	Négatif	Négatif
2017	F	plus de 6 mois	Littoral	Douala	Négatif	Négatif	Négatif	Négatif	Négatif
2017	M	plus de 6 mois	Littoral	Douala	Négatif	Négatif	Négatif	Négatif	Négatif
2017	F	plus de 6 mois	Littoral	Douala	Négatif	Négatif	Négatif	Négatif	Négatif
2017	F	plus de 6 mois	Littoral	Douala	Positif	Négatif	Positif	Négatif	Négatif
2017	F	moins de 6 mois	Littoral	Douala	Positif	Négatif	Positif	Négatif	Négatif
2017	M	plus de 6 mois	Littoral	Douala	Négatif	Négatif	Négatif	Négatif	Négatif
2017	M	moins de 6 mois	Littoral	Douala	Négatif	Négatif	Négatif	Négatif	Négatif
2018	F	plus de 6 mois	Centre	Yaoundé	Négatif	Négatif	Négatif	Négatif	Négatif
2018	F	moins de 6 mois	Centre	Yaoundé	Positif	Négatif	Positif	Négatif	Négatif
2018	F	moins de 6 mois	Centre	Yaoundé	Négatif	Positif	Positif	Négatif	Négatif
2018	F	plus de 6 mois	Centre	Yaoundé	Négatif	Positif	Positif	Négatif	Négatif
2018	M	plus de 6 mois	Centre	Yaoundé	Positif	Négatif	Positif	Négatif	Négatif
2018	F	plus de 6 mois	Centre	Yaoundé	Positif	Positif	Positif	Positif	Négatif
2018	F	plus de 6 mois	Centre	Yaoundé	Négatif	Négatif	Négatif	Négatif	Négatif
2018	M	plus de 6 mois	Centre	Yaoundé	Négatif	Positif	Positif	Négatif	Négatif
2018	F	plus de 6 mois	Centre	Yaoundé	Négatif	Positif	Positif	Négatif	Négatif
2018	M	plus de 6 mois	Centre	Yaoundé	Négatif	Négatif	Négatif	Négatif	Négatif
2018	F	plus de 6 mois	Centre	Yaoundé	Positif	Négatif	Positif	Négatif	Négatif
2018	F	plus de 6 mois	Centre	Yaoundé	Négatif	Négatif	Négatif	Négatif	Négatif
2018	M	plus de 6 mois	Centre	Yaoundé	Positif	Négatif	Positif	Négatif	Négatif
2018	M	plus de 6 mois	Centre	Yaoundé	Négatif	Négatif	Négatif	Négatif	Négatif
2018	F	plus de 6 mois	Centre	Yaoundé	Négatif	Négatif	Négatif	Négatif	Négatif
2018	F	plus de 6 mois	Centre	Yaoundé	Négatif	Négatif	Négatif	Négatif	Négatif
2018	F	plus de 6 mois	Centre	Yaoundé	Négatif	Négatif	Négatif	Négatif	Négatif
2018	F	plus de 6 mois	Centre	Yaoundé	Négatif	Négatif	Négatif	Négatif	Négatif
2018	F	plus de 6 mois	Centre	Yaoundé	Négatif	Négatif	Négatif	Négatif	Négatif
2018	F	plus de 6 mois	Centre	Yaoundé	Négatif	Négatif	Négatif	Négatif	Négatif
2018	M	plus de 6 mois	Centre	Yaoundé	Négatif	Négatif	Négatif	Négatif	Négatif
2018	M	plus de 6 mois	Centre	Yaoundé	Négatif	Négatif	Négatif	Négatif	Négatif
2018	F	moins de 6 mois	Centre	Yaoundé	Négatif	Négatif	Négatif	Négatif	Négatif
2018	F	plus de 6 mois	Centre	Yaoundé	Négatif	Négatif	Négatif	Négatif	Négatif
2018	M	plus de 6 mois	Centre	Yaoundé	Négatif	Négatif	Négatif	Négatif	Négatif

2018	F	plus de 6 mois	Centre	Yaoundé	Négatif	Négatif	Négatif	Négatif	Négatif
2018	M	plus de 6 mois	Centre	Yaoundé	Négatif	Négatif	Négatif	Négatif	Négatif
2018	F	plus de 6 mois	Centre	Yaoundé	Négatif	Négatif	Négatif	Négatif	Négatif
2018	F	plus de 6 mois	Centre	Yaoundé	Négatif	Négatif	Négatif	Négatif	Négatif
2018	F	plus de 6 mois	Centre	Yaoundé	Négatif	Négatif	Négatif	Négatif	Négatif
2018	F	plus de 6 mois	Centre	Yaoundé	Négatif	Négatif	Négatif	Négatif	Négatif
2018	F	plus de 6 mois	Centre	Yaoundé	Négatif	Négatif	Négatif	Négatif	Négatif
2018	F	plus de 6 mois	Centre	Yaoundé	Négatif	Négatif	Négatif	Négatif	Négatif
2018	F	plus de 6 mois	Centre	Yaoundé	Négatif	Négatif	Négatif	Négatif	Négatif
2018	M	plus de 6 mois	Centre	Yaoundé	Négatif	Négatif	Négatif	Négatif	Négatif
2018	F	plus de 6 mois	Centre	Yaoundé	Négatif	Négatif	Négatif	Négatif	Négatif
2018	F	plus de 6 mois	Centre	Yaoundé	Négatif	Négatif	Négatif	Négatif	Négatif
2018	M	plus de 6 mois	Centre	Yaoundé	Négatif	Négatif	Négatif	Négatif	Négatif
2018	M	plus de 6 mois	Centre	Yaoundé	Négatif	Négatif	Négatif	Négatif	Négatif
2018	F	plus de 6 mois	Centre	Yaoundé	Négatif	Négatif	Négatif	Négatif	Négatif
2018	F	plus de 6 mois	Centre	Yaoundé	Négatif	Négatif	Négatif	Négatif	Négatif
2018	F	plus de 6 mois	Centre	Yaoundé	Négatif	Négatif	Négatif	Négatif	Négatif
2018	M	plus de 6 mois	Centre	Yaoundé	Négatif	Négatif	Négatif	Négatif	Négatif
2018	M	plus de 6 mois	Centre	Yaoundé	Négatif	Négatif	Négatif	Négatif	Négatif
2018	F	plus de 6 mois	Centre	Yaoundé	Négatif	Négatif	Négatif	Négatif	Négatif
2018	M	plus de 6 mois	Centre	Yaoundé	Négatif	Négatif	Négatif	Négatif	Négatif
2018	F	plus de 6 mois	Centre	Yaoundé	Négatif	Négatif	Négatif	Négatif	Négatif
2018	F	plus de 6 mois	Centre	Yaoundé	Négatif	Négatif	Négatif	Négatif	Négatif
2018	F	plus de 6 mois	Centre	Yaoundé	Négatif	Négatif	Négatif	Négatif	Négatif
2018	M	plus de 6 mois	Centre	Yaoundé	Négatif	Négatif	Négatif	Négatif	Négatif
2018	F	plus de 6 mois	Centre	Yaoundé	Négatif	Négatif	Négatif	Négatif	Négatif
2018	F	plus de 6 mois	Centre	Yaoundé	Positif	Négatif	Positif	Négatif	Négatif
2018	M	plus de 6 mois	Centre	Yaoundé	Négatif	Négatif	Négatif	Négatif	Négatif
2018	M	plus de 6 mois	Centre	Yaoundé	Négatif	Négatif	Négatif	Négatif	Négatif
2018	F	plus de 6 mois	Centre	Yaoundé	Négatif	Positif	Positif	Négatif	Négatif
2018	F	plus de 6 mois	Centre	Yaoundé	Négatif	Négatif	Négatif	Négatif	Négatif
2018	F	plus de 6 mois	Centre	Yaoundé	Négatif	Négatif	Négatif	Négatif	Négatif
2018	F	plus de 6 mois	Centre	Yaoundé	Négatif	Négatif	Négatif	Négatif	Négatif
2018	F	plus de 6 mois	Centre	Yaoundé	Négatif	Négatif	Négatif	Négatif	Négatif
2018	M	plus de 6 mois	Centre	Yaoundé	Négatif	Négatif	Négatif	Négatif	Négatif
2018	M	plus de 6 mois	Centre	Yaoundé	Négatif	Négatif	Négatif	Négatif	Négatif
2018	F	plus de 6 mois	Centre	Yaoundé	Positif	Positif	Positif	Positif	Positif
2018	F	plus de 6 mois	Centre	Yaoundé	Négatif	Positif	Positif	Négatif	Négatif
2018	F	plus de 6 mois	Centre	Yaoundé	Positif	Négatif	Positif	Négatif	Négatif
2018	F	plus de 6 mois	Centre	Yaoundé	Positif	Négatif	Positif	Négatif	Négatif
2018	F	plus de 6 mois	Centre	Yaoundé	Positif	Négatif	Positif	Négatif	Négatif
2018	F	plus de 6 mois	Centre	Yaoundé	Négatif	Négatif	Négatif	Négatif	Négatif
2018	M	plus de 6 mois	Centre	Yaoundé	Négatif	Négatif	Négatif	Négatif	Négatif
2018	M	plus de 6 mois	Centre	Yaoundé	Négatif	Négatif	Négatif	Négatif	Négatif

2018	M	plus de 6 mois	Centre	Yaoundé	Négatif	Positif	Positif	Négatif	Négatif
2018	F	plus de 6 mois	Centre	Yaoundé	Positif	Positif	Positif	Positif	Négatif
2018	F	plus de 6 mois	Centre	Yaoundé	Négatif	Négatif	Négatif	Négatif	Négatif
2018	M	plus de 6 mois	Centre	Yaoundé	Négatif	Positif	Positif	Négatif	Négatif
2018	M	plus de 6 mois	Centre	Yaoundé	Négatif	Positif	Positif	Négatif	Négatif
2018	F	plus de 6 mois	Centre	Yaoundé	Négatif	Positif	Positif	Négatif	Négatif
2018	F	plus de 6 mois	Centre	Yaoundé	Positif	Positif	Positif	Positif	Négatif
2018	F	plus de 6 mois	Centre	Yaoundé	Négatif	Négatif	Négatif	Négatif	Négatif
2018	F	plus de 6 mois	Centre	Yaoundé	Négatif	Positif	Positif	Négatif	Négatif
2018	F	plus de 6 mois	Centre	Yaoundé	Négatif	Positif	Positif	Négatif	Positif
2018	F	plus de 6 mois	Centre	Yaoundé	Positif	Négatif	Positif	Négatif	Négatif
2018	M	plus de 6 mois	Centre	Yaoundé	Négatif	Positif	Positif	Négatif	Négatif
2018	M	plus de 6 mois	Centre	Yaoundé	Négatif	Négatif	Négatif	Négatif	Négatif
2018	F	plus de 6 mois	Centre	Yaoundé	Négatif	Positif	Positif	Négatif	Négatif
2018	M	plus de 6 mois	Centre	Yaoundé	Négatif	Positif	Positif	Négatif	Négatif
2018	F	plus de 6 mois	Centre	Yaoundé	Négatif	Positif	Positif	Négatif	Négatif
2018	F	plus de 6 mois	Centre	Yaoundé	Négatif	Positif	Positif	Négatif	Positif
2018	F	plus de 6 mois	Centre	Yaoundé	Positif	Négatif	Positif	Négatif	Négatif
2018	F	plus de 6 mois	Centre	Yaoundé	Négatif	Positif	Positif	Négatif	Négatif
2018	F	plus de 6 mois	Centre	Yaoundé	Négatif	Positif	Positif	Négatif	Positif
2018	F	plus de 6 mois	Centre	Yaoundé	Négatif	Négatif	Négatif	Négatif	Négatif
2018	F	plus de 6 mois	Centre	Yaoundé	Négatif	Positif	Positif	Négatif	Négatif
2018	M	plus de 6 mois	Centre	Yaoundé	Positif	Positif	Positif	Positif	Négatif
2018	F	plus de 6 mois	Centre	Yaoundé	Négatif	Positif	Positif	Négatif	Négatif
2018	F	plus de 6 mois	Centre	Yaoundé	Négatif	Positif	Positif	Négatif	Négatif
2018	M	plus de 6 mois	Centre	Yaoundé	Négatif	Positif	Positif	Négatif	Négatif
2018	F	plus de 6 mois	Centre	Yaoundé	Négatif	Positif	Positif	Négatif	Négatif
2018	M	plus de 6 mois	Centre	Yaoundé	Négatif	Négatif	Négatif	Négatif	Négatif
2018	M	plus de 6 mois	Centre	Yaoundé	Positif	Positif	Positif	Positif	Négatif
2018	F	plus de 6 mois	Centre	Yaoundé	Négatif	Négatif	Négatif	Négatif	Négatif
2018	F	plus de 6 mois	Centre	Yaoundé	Négatif	Positif	Positif	Négatif	Négatif
2018	F	plus de 6 mois	Centre	Yaoundé	Négatif	Positif	Positif	Négatif	Négatif
2018	F	plus de 6 mois	Centre	Yaoundé	Positif	Positif	Positif	Positif	Négatif
2018	F	plus de 6 mois	Centre	Yaoundé	Positif	Négatif	Positif	Négatif	Négatif
2018	F	plus de 6 mois	Centre	Yaoundé	Négatif	Négatif	Négatif	Négatif	Négatif
2018	F	plus de 6 mois	Centre	Yaoundé	Négatif	Positif	Positif	Négatif	Négatif
2018	F	plus de 6 mois	Centre	Yaoundé	Positif	Positif	Positif	Positif	Négatif
2018	M	plus de 6 mois	Centre	Yaoundé	Positif	Négatif	Positif	Négatif	Négatif
2018	M	plus de 6 mois	Centre	Yaoundé	Négatif	Négatif	Négatif	Négatif	Négatif
2018	F	plus de 6 mois	Centre	Yaoundé	Négatif	Positif	Positif	Négatif	Négatif
2018	F	plus de 6 mois	Centre	Yaoundé	Positif	Négatif	Positif	Négatif	Négatif
2018	M	plus de 6 mois	Centre	Yaoundé	Négatif	Négatif	Négatif	Négatif	Négatif
2018	F	plus de 6 mois	Centre	Yaoundé	Positif	Négatif	Positif	Négatif	Négatif
2018	F	plus de 6 mois	Centre	Yaoundé	Négatif	Négatif	Négatif	Négatif	Négatif

2018	M	plus de 6 mois	Centre	Yaoundé	Positif	Négatif	Positif	Négatif	Négatif
2018	M	plus de 6 mois	Centre	Yaoundé	Positif	Négatif	Positif	Négatif	Négatif
2018	M	plus de 6 mois	Centre	Yaoundé	Négatif	Négatif	Négatif	Négatif	Négatif
2018	F	plus de 6 mois	Centre	Yaoundé	Positif	Positif	Positif	Positif	Négatif
2018	M	plus de 6 mois	Centre	Yaoundé	Négatif	Positif	Positif	Négatif	Positif
2018	M	plus de 6 mois	Centre	Yaoundé	Positif	Négatif	Positif	Négatif	Négatif
2018	F	plus de 6 mois	Centre	Yaoundé	Négatif	Négatif	Négatif	Négatif	Négatif
2018	F	plus de 6 mois	Centre	Yaoundé	Négatif	Positif	Positif	Négatif	Négatif
2018	M	plus de 6 mois	Centre	Yaoundé	Positif	Positif	Positif	Positif	Négatif
2018	F	plus de 6 mois	Centre	Yaoundé	Négatif	Négatif	Négatif	Négatif	Négatif
2018	M	plus de 6 mois	Centre	Yaoundé	Négatif	Positif	Positif	Négatif	Négatif
2018	M	plus de 6 mois	Centre	Yaoundé	Négatif	Négatif	Négatif	Négatif	Négatif
2018	F	plus de 6 mois	Centre	Yaoundé	Négatif	Positif	Positif	Négatif	Négatif
2018	F	plus de 6 mois	Centre	Yaoundé	Positif	Positif	Positif	Positif	Négatif
2018	F	plus de 6 mois	Centre	Yaoundé	Négatif	Négatif	Négatif	Négatif	Négatif
2018	M	plus de 6 mois	Centre	Yaoundé	Négatif	Positif	Positif	Négatif	Négatif
2018	M	plus de 6 mois	Centre	Yaoundé	Positif	Positif	Positif	Positif	Négatif
2018	M	plus de 6 mois	Centre	Yaoundé	Négatif	Positif	Positif	Négatif	Négatif
2018	F	plus de 6 mois	Centre	Yaoundé	Négatif	Négatif	Négatif	Négatif	Négatif
2018	M	plus de 6 mois	Centre	Yaoundé	Négatif	Positif	Positif	Négatif	Positif
2018	F	plus de 6 mois	Centre	Yaoundé	Négatif	Négatif	Négatif	Négatif	Négatif
2018	F	plus de 6 mois	Centre	Yaoundé	Négatif	Négatif	Négatif	Négatif	Négatif
2018	F	plus de 6 mois	Centre	Yaoundé	Positif	Négatif	Positif	Négatif	Négatif
2018	M	plus de 6 mois	Centre	Yaoundé	Positif	Négatif	Positif	Négatif	Négatif
2018	F	moins de 6 mois	Centre	Yaoundé	Négatif	Négatif	Négatif	Négatif	Négatif
2018	F	moins de 6 mois	Centre	Yaoundé	Positif	Positif	Positif	Positif	Négatif
2018	F	moins de 6 mois	Centre	Yaoundé	Positif	Négatif	Positif	Négatif	Négatif
2018	F	plus de 6 mois	Centre	Yaoundé	Négatif	Positif	Positif	Négatif	Négatif
2018	F	plus de 6 mois	Centre	Yaoundé	Positif	Négatif	Positif	Négatif	Négatif
2018	F	plus de 6 mois	Centre	Yaoundé	Négatif	Positif	Positif	Négatif	Négatif
2018	F	moins de 6 mois	Centre	Yaoundé	Négatif	Négatif	Négatif	Négatif	Négatif
2018	M	plus de 6 mois	Centre	Yaoundé	Positif	Négatif	Positif	Négatif	Négatif
2018	F	plus de 6 mois	Centre	Yaoundé	Positif	Négatif	Positif	Négatif	Négatif
2018	M	plus de 6 mois	Centre	Yaoundé	Négatif	Positif	Positif	Négatif	Positif
2018	F	plus de 6 mois	Centre	Yaoundé	Positif	Négatif	Positif	Négatif	Négatif
2018	F	plus de 6 mois	Centre	Yaoundé	Positif	Négatif	Positif	Négatif	Négatif
2018	M	plus de 6 mois	Centre	Yaoundé	Positif	Positif	Positif	Positif	Négatif
2018	M	plus de 6 mois	Centre	Yaoundé	Négatif	Négatif	Négatif	Négatif	Négatif
2018	M	plus de 6 mois	Centre	Yaoundé	Positif	Négatif	Positif	Négatif	Négatif
2018	F	plus de 6 mois	Centre	Yaoundé	Négatif	Négatif	Négatif	Négatif	Négatif
2018	M	plus de 6 mois	Centre	Yaoundé	Positif	Négatif	Positif	Négatif	Négatif
2018	F	plus de 6 mois	Centre	Yaoundé	Négatif	Négatif	Négatif	Négatif	Négatif
2018	F	moins de 6 mois	Centre	Yaoundé	Négatif	Négatif	Négatif	Négatif	Négatif
2018	M	plus de 6 mois	Centre	Yaoundé	Positif	Négatif	Positif	Négatif	Négatif

2018	F	plus de 6 mois	Centre	Yaoundé	Positif	Positif	Positif	Positif	Négatif
2018	F	plus de 6 mois	Centre	Yaoundé	Négatif	Positif	Positif	Négatif	Négatif
2018	M	moins de 6 mois	Centre	Yaoundé	Négatif	Positif	Positif	Négatif	Négatif
2018	M	moins de 6 mois	Centre	Yaoundé	Négatif	Négatif	Négatif	Négatif	Négatif
2018	M	plus de 6 mois	Centre	Yaoundé	Positif	Négatif	Positif	Négatif	Négatif
2018	M	plus de 6 mois	Centre	Yaoundé	Négatif	Négatif	Négatif	Négatif	Négatif
2018	M	plus de 6 mois	Centre	Yaoundé	Négatif	Négatif	Négatif	Négatif	Négatif
2018	F	plus de 6 mois	Centre	Yaoundé	Négatif	Positif	Positif	Négatif	Négatif
2018	F	plus de 6 mois	Centre	Yaoundé	Négatif	Négatif	Négatif	Négatif	Négatif
2018	F	plus de 6 mois	Centre	Yaoundé	Négatif	Négatif	Négatif	Négatif	Négatif
2018	F	plus de 6 mois	Centre	Yaoundé	Positif	Négatif	Positif	Négatif	Négatif
2018	F	plus de 6 mois	Centre	Yaoundé	Positif	Négatif	Positif	Négatif	Négatif
2018	M	plus de 6 mois	Centre	Yaoundé	Négatif	Négatif	Négatif	Négatif	Négatif
2018	F	plus de 6 mois	Centre	Yaoundé	Négatif	Négatif	Négatif	Négatif	Négatif
2018	F	plus de 6 mois	Centre	Yaoundé	Négatif	Positif	Positif	Négatif	Négatif
2018	F	plus de 6 mois	Centre	Yaoundé	Négatif	Positif	Positif	Négatif	Négatif
2018	F	moins de 6 mois	Centre	Yaoundé	Positif	Négatif	Positif	Négatif	Négatif
2018	M	plus de 6 mois	Centre	Yaoundé	Positif	Négatif	Positif	Négatif	Négatif
2018	M	moins de 6 mois	Centre	Yaoundé	Négatif	Négatif	Négatif	Négatif	Négatif
2018	F	plus de 6 mois	Centre	Yaoundé	Négatif	Négatif	Négatif	Négatif	Négatif
2018	F	plus de 6 mois	Centre	Yaoundé	Positif	Négatif	Positif	Négatif	Négatif
2018	M	moins de 6 mois	Centre	Yaoundé	Négatif	Négatif	Négatif	Négatif	Négatif
2018	F	plus de 6 mois	Centre	Yaoundé	Négatif	Positif	Positif	Négatif	Négatif
2018	M	plus de 6 mois	Centre	Yaoundé	Négatif	Positif	Positif	Négatif	Négatif
2018	F	moins de 6 mois	Centre	Yaoundé	Positif	Positif	Positif	Positif	Négatif
2018	F	plus de 6 mois	Centre	Yaoundé	Positif	Négatif	Positif	Négatif	Négatif
2018	F	plus de 6 mois	Centre	Yaoundé	Négatif	Négatif	Négatif	Négatif	Négatif
2018	M	moins de 6 mois	Centre	Yaoundé	Positif	Négatif	Positif	Négatif	Négatif
2018	F	plus de 6 mois	Centre	Yaoundé	Négatif	Négatif	Négatif	Négatif	Négatif
2018	F	plus de 6 mois	Centre	Yaoundé	Positif	Positif	Positif	Positif	Négatif
2018	F	moins de 6 mois	Centre	Yaoundé	Positif	Positif	Positif	Positif	Négatif
2018	M	plus de 6 mois	Centre	Yaoundé	Négatif	Négatif	Négatif	Négatif	Négatif
2018	F	plus de 6 mois	Centre	Yaoundé	Positif	Positif	Positif	Positif	Négatif
2018	M	plus de 6 mois	Centre	Yaoundé	Positif	Négatif	Positif	Négatif	Négatif
2018	F	moins de 6 mois	Centre	Yaoundé	Positif	Positif	Positif	Positif	Négatif
2018	F	plus de 6 mois	Centre	Yaoundé	Positif	Positif	Positif	Positif	Positif
2018	F	plus de 6 mois	Centre	Yaoundé	Positif	Négatif	Positif	Négatif	Négatif
2018	F	plus de 6 mois	Centre	Yaoundé	Négatif	Positif	Positif	Négatif	Négatif
2018	M	plus de 6 mois	Centre	Yaoundé	Négatif	Positif	Positif	Négatif	Négatif
2018	M	plus de 6 mois	Centre	Yaoundé	Négatif	Positif	Positif	Négatif	Négatif
2018	F	moins de 6 mois	Centre	Yaoundé	Positif	Négatif	Positif	Négatif	Négatif
2018	F	plus de 6 mois	Centre	Yaoundé	Positif	Positif	Positif	Positif	Négatif
2018	F	plus de 6 mois	Centre	Yaoundé	Positif	Positif	Positif	Positif	Négatif
2018	M	plus de 6 mois	Centre	Yaoundé	Positif	Négatif	Positif	Négatif	Négatif

2018	F	plus de 6 mois	Centre	Yaoundé	Négatif	Négatif	Négatif	Négatif	Négatif
2018	F	moins de 6 mois	Centre	Yaoundé	Négatif	Négatif	Négatif	Négatif	Négatif
2018	F	plus de 6 mois	Centre	Yaoundé	Négatif	Négatif	Négatif	Négatif	Négatif
2018	F	plus de 6 mois	Centre	Yaoundé	Négatif	Négatif	Négatif	Négatif	Négatif
2018	F	moins de 6 mois	Centre	Yaoundé	Négatif	Positif	Positif	Négatif	Négatif
2018	F	moins de 6 mois	Centre	Yaoundé	Négatif	Négatif	Négatif	Négatif	Négatif
2018	M	plus de 6 mois	Centre	Yaoundé	Négatif	Négatif	Négatif	Négatif	Négatif
2018	F	moins de 6 mois	Centre	Yaoundé	Positif	Négatif	Positif	Négatif	Négatif
2018	M	moins de 6 mois	Centre	Yaoundé	Négatif	Négatif	Négatif	Négatif	Négatif
2018	F	plus de 6 mois	Centre	Yaoundé	Positif	Positif	Positif	Positif	Négatif
2018	F	moins de 6 mois	Centre	Yaoundé	Positif	Positif	Positif	Positif	Négatif
2018	M	plus de 6 mois	Centre	Yaoundé	Positif	Positif	Positif	Positif	Négatif
2018	F	moins de 6 mois	Centre	Yaoundé	Négatif	Négatif	Négatif	Négatif	Négatif
2018	M	moins de 6 mois	Centre	Yaoundé	Négatif	Négatif	Négatif	Négatif	Négatif
2018	F	plus de 6 mois	Centre	Yaoundé	Positif	Positif	Positif	Positif	Négatif
2018	F	plus de 6 mois	Centre	Yaoundé	Négatif	Positif	Positif	Négatif	Négatif
2018	F	moins de 6 mois	Centre	Yaoundé	Négatif	Positif	Positif	Négatif	Négatif
2018	F	plus de 6 mois	Centre	Yaoundé	Négatif	Positif	Positif	Négatif	Négatif
2018	M	plus de 6 mois	Centre	Yaoundé	Positif	Négatif	Positif	Négatif	Négatif
2018	F	plus de 6 mois	Centre	Yaoundé	Négatif	Négatif	Négatif	Négatif	Négatif
2018	M	plus de 6 mois	Centre	Yaoundé	Positif	Négatif	Positif	Négatif	Négatif
2018	M	moins de 6 mois	Centre	Yaoundé	Négatif	Positif	Positif	Négatif	Négatif
2018	M	plus de 6 mois	Centre	Yaoundé	Positif	Négatif	Positif	Négatif	Négatif
2018	F	plus de 6 mois	Centre	Yaoundé	Négatif	Positif	Positif	Négatif	Négatif
2018	F	moins de 6 mois	Centre	Yaoundé	Positif	Positif	Positif	Positif	Négatif
2018	F	plus de 6 mois	Centre	Yaoundé	Positif	Négatif	Positif	Négatif	Négatif
2018	M	moins de 6 mois	Centre	Yaoundé	Négatif	Positif	Positif	Négatif	Négatif
2018	M	moins de 6 mois	Centre	Yaoundé	Négatif	Positif	Positif	Négatif	Négatif
2018	M	plus de 6 mois	Centre	Yaoundé	Positif	Négatif	Positif	Négatif	Négatif
2018	F	plus de 6 mois	Centre	Yaoundé	Négatif	Positif	Positif	Négatif	Négatif
2018	M	moins de 6 mois	Centre	Yaoundé	Négatif	Positif	Positif	Négatif	Négatif
2018	M	moins de 6 mois	Centre	Yaoundé	Négatif	Négatif	Négatif	Négatif	Négatif
2018	F	plus de 6 mois	Centre	Yaoundé	Positif	Positif	Positif	Positif	Négatif
2018	M	moins de 6 mois	Centre	Yaoundé	Négatif	Négatif	Négatif	Négatif	Négatif
2018	M	plus de 6 mois	Centre	Yaoundé	Positif	Positif	Positif	Positif	Négatif
2018	F	plus de 6 mois	Centre	Yaoundé	Positif	Négatif	Positif	Négatif	Négatif
2018	F	plus de 6 mois	Centre	Yaoundé	Positif	Négatif	Positif	Négatif	Négatif
2018	M	moins de 6 mois	Centre	Yaoundé	Positif	Négatif	Positif	Négatif	Négatif
2018	M	plus de 6 mois	Centre	Yaoundé	Négatif	Négatif	Négatif	Négatif	Négatif
2018	F	plus de 6 mois	Centre	Yaoundé	Négatif	Négatif	Négatif	Négatif	Négatif
2018	F	plus de 6 mois	Centre	Yaoundé	Négatif	Positif	Positif	Négatif	Négatif
2018	M	moins de 6 mois	Centre	Yaoundé	Positif	Négatif	Positif	Négatif	Négatif
2018	M	moins de 6 mois	Centre	Yaoundé	Positif	Positif	Positif	Positif	Négatif
2018	F	moins de 6 mois	Centre	Yaoundé	Positif	Positif	Positif	Positif	Négatif

2018	F	plus de 6 mois	Centre	Yaoundé	Positif	Positif	Positif	Positif	Négatif
2018	M	moins de 6 mois	Centre	Yaoundé	Positif	Négatif	Positif	Négatif	Négatif
2018	M	plus de 6 mois	Centre	Yaoundé	Négatif	Négatif	Négatif	Négatif	Négatif
2018	F	plus de 6 mois	Centre	Yaoundé	Positif	Négatif	Positif	Négatif	Négatif
2018	F	plus de 6 mois	Centre	Yaoundé	Positif	Positif	Positif	Positif	Négatif
2018	M	plus de 6 mois	Centre	Yaoundé	Négatif	Négatif	Négatif	Négatif	Négatif
2018	M	moins de 6 mois	Centre	Yaoundé	Négatif	Positif	Positif	Négatif	Négatif
2018	F	plus de 6 mois	Centre	Yaoundé	Négatif	Négatif	Négatif	Négatif	Négatif
2018	F	plus de 6 mois	Centre	Yaoundé	Négatif	Négatif	Négatif	Négatif	Négatif
2018	M	plus de 6 mois	Centre	Yaoundé	Négatif	Négatif	Négatif	Négatif	Négatif
2018	F	plus de 6 mois	Centre	Yaoundé	Négatif	Négatif	Négatif	Négatif	Négatif
2018	F	plus de 6 mois	Centre	Yaoundé	Négatif	Négatif	Négatif	Négatif	Négatif
2018	M	plus de 6 mois	Centre	Yaoundé	Négatif	Négatif	Négatif	Négatif	Négatif
2018	M	plus de 6 mois	Centre	Yaoundé	Négatif	Négatif	Négatif	Négatif	Négatif
2018	M	plus de 6 mois	Centre	Yaoundé	Négatif	Négatif	Négatif	Négatif	Négatif
2018	M	moins de 6 mois	Centre	Yaoundé	Négatif	Négatif	Négatif	Négatif	Négatif
2018	F	plus de 6 mois	Centre	Yaoundé	Positif	Négatif	Positif	Négatif	Négatif
2018	F	plus de 6 mois	Centre	Yaoundé	Positif	Négatif	Positif	Négatif	Négatif
2018	F	plus de 6 mois	Centre	Yaoundé	Négatif	Négatif	Négatif	Négatif	Négatif
2018	M	plus de 6 mois	Centre	Yaoundé	Négatif	Positif	Positif	Négatif	Négatif
2018	M	plus de 6 mois	Centre	Yaoundé	Négatif	Négatif	Négatif	Négatif	Négatif
2018	F	plus de 6 mois	Centre	Yaoundé	Négatif	Négatif	Négatif	Négatif	Négatif
2018	F	plus de 6 mois	Centre	Yaoundé	Négatif	Négatif	Négatif	Négatif	Négatif
2018	F	moins de 6 mois	Centre	Yaoundé	Positif	Négatif	Positif	Négatif	Négatif
2018	F	moins de 6 mois	Centre	Yaoundé	Négatif	Négatif	Négatif	Négatif	Négatif
2018	M	plus de 6 mois	Centre	Yaoundé	Positif	Négatif	Positif	Négatif	Négatif
2018	F	plus de 6 mois	Centre	Yaoundé	Positif	Négatif	Positif	Négatif	Négatif
2018	F	plus de 6 mois	Centre	Yaoundé	Positif	Positif	Positif	Positif	Négatif
2018	F	moins de 6 mois	Centre	Yaoundé	Positif	Négatif	Positif	Négatif	Négatif
2018	M	plus de 6 mois	Centre	Yaoundé	Négatif	Positif	Positif	Négatif	Négatif
2018	F	moins de 6 mois	Centre	Yaoundé	Négatif	Positif	Positif	Négatif	Négatif
2018	M	plus de 6 mois	Centre	Yaoundé	Négatif	Positif	Positif	Négatif	Négatif
2018	M	plus de 6 mois	Centre	Yaoundé	Positif	Positif	Positif	Positif	Négatif
2018	F	plus de 6 mois	Centre	Yaoundé	Négatif	Positif	Positif	Négatif	Négatif
2018	F	moins de 6 mois	Centre	Yaoundé	Négatif	Négatif	Négatif	Négatif	Négatif
2018	F	moins de 6 mois	Centre	Yaoundé	Négatif	Négatif	Négatif	Négatif	Négatif
2018	F	plus de 6 mois	Centre	Yaoundé	Négatif	Négatif	Négatif	Négatif	Négatif
2018	F	plus de 6 mois	Centre	Yaoundé	Positif	Négatif	Positif	Négatif	Négatif
2018	F	moins de 6 mois	Centre	Yaoundé	Négatif	Négatif	Négatif	Négatif	Négatif
2018	M	moins de 6 mois	Centre	Yaoundé	Négatif	Positif	Positif	Négatif	Négatif
2018	M	moins de 6 mois	Centre	Yaoundé	Positif	Négatif	Positif	Négatif	Négatif
2018	F	moins de 6 mois	Centre	Yaoundé	Positif	Négatif	Positif	Négatif	Négatif
2018	F	plus de 6 mois	Centre	Yaoundé	Positif	Positif	Positif	Positif	Négatif
2018	F	plus de 6 mois	Centre	Yaoundé	Négatif	Positif	Positif	Négatif	Négatif

2018	F	plus de 6 mois	Centre	Yaoundé	Négatif	Négatif	Négatif	Négatif	Négatif
2018	M	moins de 6 mois	Centre	Yaoundé	Négatif	Positif	Positif	Négatif	Négatif
2018	M	moins de 6 mois	Centre	Yaoundé	Négatif	Positif	Positif	Négatif	Négatif
2018	M	moins de 6 mois	Centre	Yaoundé	Négatif	Positif	Positif	Négatif	Négatif
2018	F	moins de 6 mois	Centre	Yaoundé	Négatif	Positif	Positif	Négatif	Négatif
2018	M	plus de 6 mois	Centre	Yaoundé	Positif	Positif	Positif	Positif	Négatif
2018	M	plus de 6 mois	Centre	Yaoundé	Négatif	Positif	Positif	Négatif	Négatif
2018	F	moins de 6 mois	Centre	Yaoundé	Négatif	Positif	Positif	Négatif	Négatif
2018	F	plus de 6 mois	Centre	Yaoundé	Négatif	Négatif	Négatif	Négatif	Négatif
2018	F	plus de 6 mois	Centre	Yaoundé	Négatif	Négatif	Négatif	Négatif	Négatif
2018	F	plus de 6 mois	Centre	Yaoundé	Positif	Négatif	Positif	Négatif	Négatif
2018	F	moins de 6 mois	Centre	Yaoundé	Négatif	Négatif	Négatif	Négatif	Négatif
2018	M	moins de 6 mois	Centre	Yaoundé	Négatif	Positif	Positif	Négatif	Négatif
2018	F	moins de 6 mois	Centre	Yaoundé	Négatif	Positif	Positif	Négatif	Négatif
